# A novel nomogram predicting the risk of positive biopsy for patients in the diagnostic gray area of prostate cancer

**DOI:** 10.1038/s41598-020-74703-8

**Published:** 2020-10-19

**Authors:** Guang-Dong Hou, Yu Zheng, Wan-Xiang Zheng, Ming Gao, Lei Zhang, Niu-Niu Hou, Jia-Rui Yuan, Di Wei, Dong-En Ju, Xin-Long Dun, Fu-Li Wang, Jian-Lin Yuan

**Affiliations:** 1 Department of Urology, Xijing Hospital, Fourth Military Medical University, Xi’an, 710032 China; 2 Department of Andrology, Xi’an Daxing Hospital, Shaanxi University of Chinese Medicine, Xi’an, 710016 China; 3 Department of Thyroid, Breast and Vascular Surgery, Xijing Hospital, Fourth Military Medical University, Xi’an, 710032 China; 4 St. George’s University School of Medicine, True Blue, Grenada

**Keywords:** Oncology, Urology

## Abstract

The roles played by several inflammatory factors in screening for prostate cancer (PCa) among gray area patients, namely those with serum prostate-specific antigen (PSA) levels between 4 and 10 ng/ml, have not been completely identified, and few effective diagnostic nomograms have been developed exclusively for these patients. We aimed to investigate new independent predictors of positive biopsy (PB) results and develop a novel diagnostic nomogram for this group of patients. The independent predictors of PB results were identified, and a nomogram was constructed using multivariate logistic regression analysis based on a cohort comprising 401 Gy area patients diagnosed at Xijing Hospital (Xi’an, China) between January 2016 and December 2019. The predictive accuracy of the nomogram was assessed using the receiver operating characteristic curve, and the nomogram was calibrated by comparing the prediction with the observation. The performance of the nomogram was further validated using an independent cohort. Finally, lymphocyte-to-monocyte ratio (LMR) > 4.11 and red blood cell distribution width (RDW)-standard deviation (SD) > 42.9 fl were identified as independent protective predictors of PB results, whereas PSA density (PSAD) > 0.141 was identified as an independent risk predictor. The nomogram established using PSAD, LMR, and RDW-SD was perfectly calibrated, and its predictive accuracy was superior to that of PSAD in both internal and external validations (0.827 vs 0.769 and 0.765 vs 0.713, respectively). This study is the first to report the importance of LMR and RDW-SD in screening for PCa among gray area patients and to construct an exclusive nomogram to predict the individual risk of positive 13-core biopsy results in this group of patients. With superior performance over PSAD, our nomogram will help increase the accuracy of PCa screening, thereby avoiding unnecessary biopsy.

## Introduction

With approximately 1.3 million newly diagnosed cases and approximately 359,000 deaths in 2018, prostate cancer (PCa) is the second most prevalent malignancy worldwide and the fifth leading cancer-associated cause of death in men^1^. The incidence of PCa in China is on the rise^2^. Transrectal ultrasound-guided prostate biopsy remains the gold standard for the diagnosis of PCa, but it can sometimes lead to severe complications, such as hematospermia, hematuria, and sepsis^3,4^.

Serum prostate-specific antigen (PSA) is an important biomarker for screening patients suspected to have PCa. Serum PSA levels > 4 ng/ml are universally accepted to be an indication for prostate biopsy. However, the rate of positive biopsy (PB) results is < 20% among Chinese patients with serum PSA levels between 4 and 10 ng/ml^5^, who are widely considered to be in the diagnostic “gray area” for PCa. For these gray area patients, the 2014 Chinese Urological Guidelines strongly recommend considering free PSA (fPSA)/total PSA (tPSA) and PSA density (PSAD) evaluation to determine whether a biopsy should be performed. Recently, several imaging modalities and biomarkers have been suggested to increase the accuracy of screening for PCa among gray area patients, but few of them are available for widespread use because of high costs or lack of standardization and regional availability.

Currently, the roles played by serum pretreatment neutrophil-to-lymphocyte ratio (NLR), lymphocyte-to-monocyte ratio (LMR), platelet-to-lymphocyte ratio (PLR), and red blood cell distribution width (RDW)-standard deviation (SD) in screening for PCa among gray area patients are either controversial or unclear, and some of these parameters have not been investigated in Chinese gray area patients. In terms of diagnostic nomograms to predict the risk of PCa, only one nomogram is based exclusively on the data of gray area patients, ie, the “Zhu nomogram”^5^. The Zhu nomogram, however, has considerable limitations in clinical practice because it is based on data from patients who underwent biopsy without a uniform core, which inevitably decreases the predictive accuracy.

To address the above concerns, the present study aimed to clarify whether NLR, LMR, PLR, and RDW-SD can serve as independent predictors of PB results in gray area patients and develop a novel nomogram to predict the individual risk of PB results exclusive to this group of patients.

## Patients and methods

### Study population

A total of 401 patients with serum PSA levels between 4 and 10 ng/ml who were registered at Xijing Hospital (Xi’an, China) between January 2016 and December 2019 constituted the training cohort that was used to develop the nomogram. All the patients met the following inclusion criteria: (1) undergoing prostate biopsy for the first time, (2) three-dimensional size of the prostate available via transabdominal ultrasonography before biopsy, (3) blood tests performed within 1 week before biopsy, (4) complete clinical and pathological data available, (5) absence of acute prostatitis or systemic inflammatory disease, (6) absence of urinary tract infection, (7) no history of prostate surgery, (8) no history of 5-alpha reductase inhibitor use, and (9) no anti-inflammatory drug use within 2 weeks before blood tests. In addition, an independent validation cohort comprising 276 patients diagnosed at the same center between January 2013 and December 2015 was used to externally validate the nomogram. Patients in the validation cohort met the same inclusion criteria as those in the training cohort.

All the study patients provided written informed consent. All procedures performed in this study complied with the 1964 Helsinki Declaration and its later amendments, and all experimental protocols were approved by the Research Committees of Xijing Hospital (Xi’an, China).

### Variables included in the study

Baseline variables included age at biopsy, prostate volume (PV), fPSA/tPSA, PSAD, NLR, LMR, PLR, and RDW-SD. PVs were calculated using the modified ellipsoid formula (0.523 × length × width × height) using measurements obtained from an ultrasound image of the prostate. In addition, 13-core biopsies were performed as recommended by Eskew et al.^6^.

### Statistical analysis

All statistical tests were performed using R software (version 3.5.2; https://www.r-project.org/) and SPSS 22.0 (IBM Corp., Armonk, NY). The normality and equality of variables were assessed using Kolmogorov–Smirnov test. Normally distributed variables are expressed as the mean ± SD and nonnormally distributed variables as the median (interquartile range); the distribution of these variables between the PB and negative biopsy (NB) groups was analyzed using Student’s *t* and Mann–Whitney *U* tests, respectively. The optimal cutoff (OCF) value of variables was identified according to the maximum Youden index on the basis of the receiver operating characteristic (ROC) curves. The Spearman test was performed to assess the correlation between two continuous variables. Univariate and multivariate logistic regression analyses were performed to identify independent predictors of PB results. Then, a nomogram was constructed using the “rms” package on the basis of the multivariate logistic regression model.

The discriminatory performance, namely the predictive accuracy, of our nomogram was quantified by calculating the area under curve (AUC) of the ROC curves, with a value of 0.5 being equivalent to the toss of a coin and 1 indicating perfect prediction. Calibration curves were also plotted to assess the calibration of our nomogram, with the prediction being expected to fall on the diagonal line in a perfectly calibrated model. Differences with a two-tailed *P*-value < 0.05 were considered significant.

### Ethics approval and consent to patients

All patients who participated in this study provided written informed consent. All procedures performed in studies conformed to the 1964 Helsinki Declaration and its later amendments, and all experimental protocols were approved by the Research Committees of Xijing Hospital (Xi’an, China).

## Results

### Characteristics of variables

In addition to significant differences in PSAD (*P* < 0.001), fPSA/tPSA (*P* < 0.001), and PV (*P* < 0.001), there were significant differences in LMR (*P* < 0.001) and RDW-SD (*P* = 0.011) between the PB and NB groups in the training cohort, whereas there were no significant between-group differences in NLR (*P* = 0.079) or PLR (*P* = 0.175). Similar results were observed in the independent validation cohort (Table 1).

**Table 1:** Baseline characteristics of the training and validation cohorts.

Variables	Training set (N = 401)			Validation set (N = 276)		
	PB group (N = 78)	NB group (N = 323)	*P*-value	PB group (N = 56)	NB group (N = 220)	*P*-value
Age (years)	68.410 ± 8.067	67.879 ± 8.479	0.606	70.946 ± 8.192	68.986 ± 8.714	0.130
PV (cm^3^)	38.431 (25.544–51.170)	59.486 (43.186–79.765)	< 0.001	39.262 (27.073–56.406)	59.574 (41.710–83.025)	< 0.001
fPSA/tPSA	0.132 (0.080–0.176)	0.187 (0.141–0.247)	< 0.001	0.140 (0.094–0.177)	0.184 (0.142–0.228)	< 0.001
PSAD	0.180 (0.123–0.277)	0.101 (0.079–0.148)	< 0.001	0.174 (0.108–0.279)	0.107 (0.076–0.148)	< 0.001
NLR	2.471 (2.012–3.239)	2.275 (1.703–3.147)	0.079	2.404 (2.021–3.298)	2.234 (1.675–3.209)	0.347
LMR	3.610 (2.710–4.543)	4.278 (3.349–5.465)	< 0.001	3.583 (2.736–5.194)	4.182 (3.047–5.923)	0.025
PLR	124.9 (92.8–155.7)	113.4 (90.2–147.8)	0.175	119.6 (92.4–152.3)	107.8 (85.7–154.5)	0.112
RDW-SD (fl)	43.2 (41.5–45.8)	44.3 (42.4–46.4)	0.011	43.1 (41.2–45.8)	44.5 (42.6–46.9)	0.005

*PB* positive-biopsy, *NB* negative-biopsy, *fPSA* free/total prostate specific antigen, *tPSA* total prostate specific antigen, *PV* prostate volume, *PSAD* prostate specific antigen density, *NLR* neutrophil-to-lymphocyte ratio, *LMR* lymphocyte to monocyte ratio, *PLR* platelet to lymphocyte ratio, *RDW-SD* standard deviation of red blood cell distribution width.

PSAD showed the best discriminatory performance among all variables, with an AUC of 0.769 in the training cohort. The AUCs of PV, fPSA/tPSA, NLR, LMR, PLR, and RDW-SD in the training cohort were 0.741, 0.696, 0.564, 0.640, 0.549, and 0.593, respectively. The AUCs of PSAD, PV, fPSA/tPSA, NLR, LMR, PLR, and RDW-SD in the validation cohort were 0.713, 0.704, 0.676, 0.541, 0.597, 0.569, and 0.620, respectively. The OCF values of these variables as well as the PCa detection rates in the high-value (> OCF value) and low-value (≤ OCF value) groups for these variables are listed in Table 2.

**Table 2:** The optimal cut-off (OCF) value as well as PCa detection rates in high-value (> OCF value) group and low-value (≤ OCF value) group of variables in the training cohort.

Variables	Cut-off value	PCa detection rate		P-value
		High-value (> cut-off value) group	Low-value (≤ cut-off value) group	
PV (cm^3^)	52.509	17/223	61/178	< 0.001
PSAD	0.155	53/127	25/274	< 0.001
fPSA/tPSA	0.177	19/200	59/201	< 0.001
NLR	2.16	56/227	22/174	0.003
LMR	4.11	25/206	53/195	< 0.001
PLR	133.2	35/141	43/260	0.045
RDW-SD (fl)	42.9	40/258	38/143	0.007

*PB* positive-biopsy, *NB* negative-biopsy, *fPSA* free/total prostate specific antigen, *tPSA* total prostate specific antigen, *PV* prostate volume, *PSAD* prostate specific antigen density, *NLR* neutrophil-to-lymphocyte ratio, *LMR* lymphocyte to monocyte ratio, *PLR* platelet to lymphocyte ratio, *RDW-SD* standard deviation of red blood cell distribution width.

### Independent predictors of positive biopsy results

In univariate analysis, PV, PSAD, fPSA/tPSA, NLR, LMR, PLR, and RDW-SD were all associated with biopsy results and all achieved significance. Spearman correlation analysis showed significant correlations between fPSA/tPSA and PV (*r* = 0.457; *P* < 0.001), fPSA/tPSA and PSAD (*r* =  − 0.494; *P* < 0.001), and PV and PSAD (*r* =  − 0.845; *P* < 0.001). Therefore, NLR, LMR, PLR, RDW-SD, and PSAD were entered into subsequent multivariate analysis and fPSA/tPSA and PV were excluded. In multivariate analysis, LMR > 4.11 (odds ratio [OR] 0.346; 95% confidence interval [CI] 0.197–0.609; *P* < 0.001) and RDW-SD > 42.9 fl (OR 0.449; 95% CI 0.257–0.782;* P* = 0.005) were identified as independent protective predictors of PB results and PSAD > 0.141 (OR 6.858; 95% CI 3.900–12.060; *P* < 0.001) was found to be an independent risk predictor (Table 3).

**Table 3:** Univariate and multivariate logistic regression analysis of positive biopsy in Chinese patients with prostate specific antigen levels 4–10 ng/ml.

Variables	Univariate analysis		Multivariate analysis	
	OR (95% CI)	*P*-value	OR (95% CI)	*P*-value
PV (> 53.401 cm^3^ vs. ≤ 53.401 cm^3^)	0.173 (0.095–0.317)	< 0.001		
PSAD (> 0.141 vs. ≤ 0.141)	6.032 (3.527–10.316)	< 0.001	6.858 (3.900–12.060)	< 0.001
fPSA/tPSA (> 0.177 vs. ≤ 0.177)	0.224 (0.125–0.401)	< 0.001		
NLR (> 2.160 vs. ≤ 2.160)	2.263 (1.319–3.880)	0.003		
LMR (> 4.11 vs. ≤ 4.11)	0.405 (0.242–0.678)	0.001	0.346 (0.197–0.609)	< 0.001
PLR (> 133.2 vs. ≤ 133.2)	1.666 (1.008–2.756)	0.047		
RDW-SD (> 42.9 fl vs. ≤ 42.9 fl)	0.507 (0.307–0.837)	0.008	0.449 (0.257–0.782)	0.005

*PB* positive-biopsy, *NB* negative-biopsy, *fPSA* free/total prostate specific antigen, *tPSA* total prostate specific antigen, *PV* prostate volume, *PSAD* prostate specific antigen density, *NLR* neutrophil-to-lymphocyte ratio, *LMR* lymphocyte to monocyte ratio, *PLR* platelet to lymphocyte ratio, *RDW-SD* standard deviation of red blood cell distribution width.

### Construction and evaluation of the nomogram

**Figure 1:**
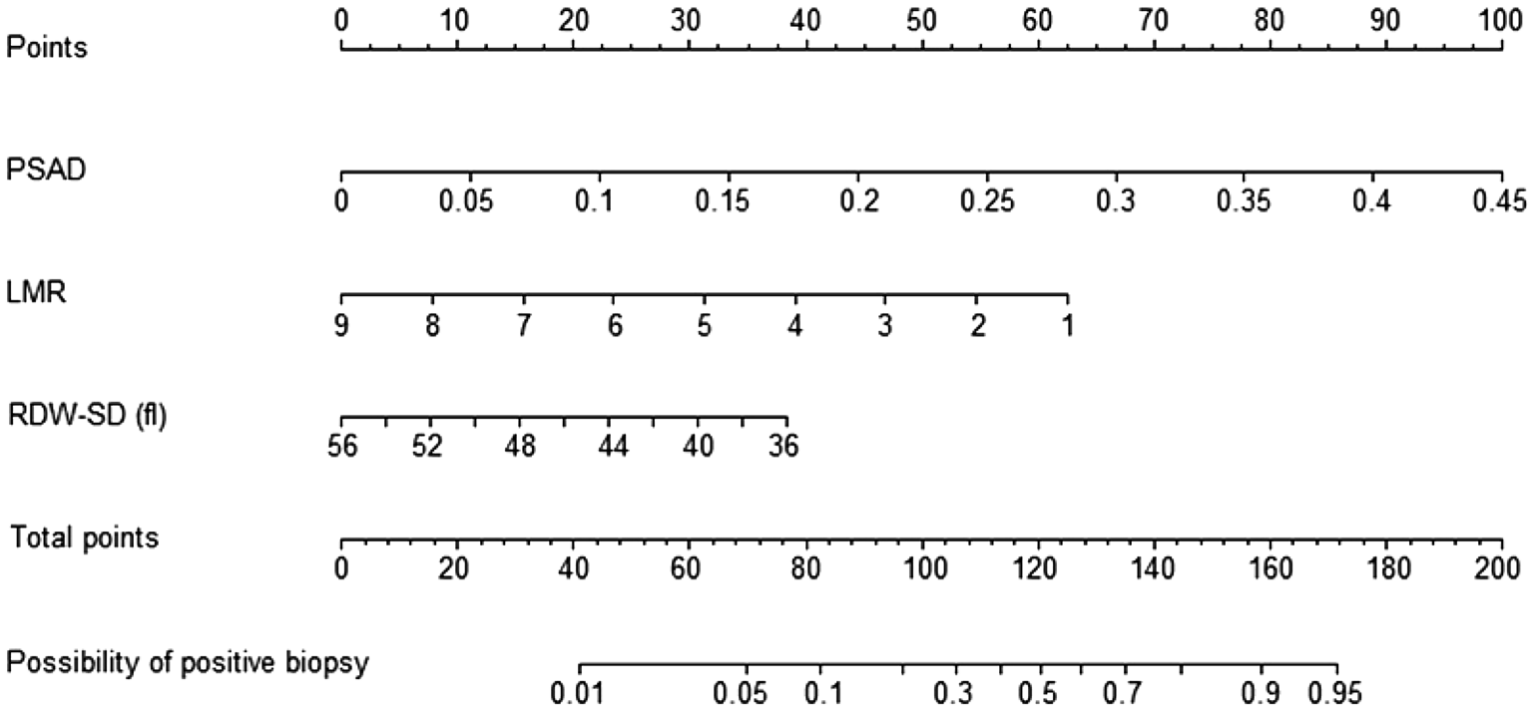
Nomogram predicting the risk of positive 13-core biopsy in patients with prostate specific antigen levels of 4–10 ng/ml.

**Figure 2:**
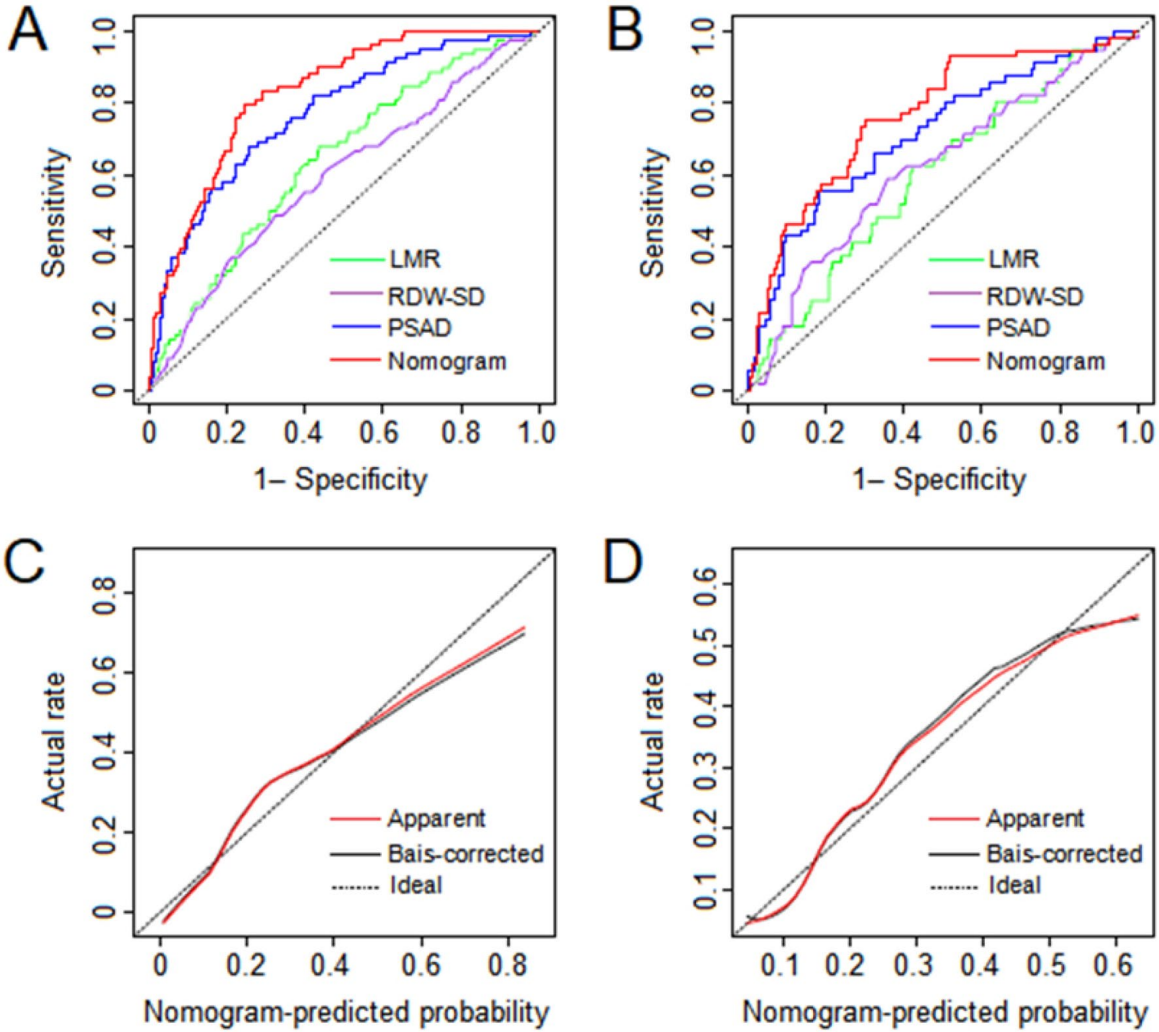
ROC curves of the nomogram in the internal (**A**) and external (**B**) validations. Calibration curves of the nomogram in the internal (**C**) and external (**D**) validations. Calibration curves depict the calibration of the nomogram in terms of agreement between the predicted possibility and actual rate of positive biopsy. The diagonal line represents a perfect prediction. The red solid line represents the apparent predictive performance of the nomogram without correction for over fit, while the black solid line represents bootstrap corrected accuracy, and the closer they fit are to the ideal line, the better the predictive performance of the nomogram is.

The nomogram was constructed by integrating PSAD, LMR, and RDW-SD according to the results of multivariate logistic regression (Fig. 1). Furthermore, the accuracy of the nomogram was assessed by comparing its predictive accuracy with that of PSAD, which made the largest contribution to biopsy results in our cohort. Our nomogram showed better predictive accuracy than PSAD in both internal (0.827 vs 0.769) and external (0.765 vs 0.713) validations (Fig. 2A,B). Moreover, the nomogram was perfectly calibrated in both internal and external validations, as the nomogram-predicted risk of PB results was in close agreement with the actual rate of PB results (Fig. 2C,D).

## Discussion

Patients with serum PSA levels between 4 and 10 ng/ml are widely considered to fall into a diagnostic gray area for PCa and are more likely to receive unnecessary biopsies. At present, the roles played by serum pretreatment NLR, LMR, PLR, and RDW-SD in screening for PCa among gray area patients are either controversial or unclear, and LMR, PLR, and RDW-SD remain to be investigated among Chinese gray area patients. Thus far, the only diagnostic nomogram established using data of only gray area patients has considerable limitations in clinical practice. Hence, the objectives of the present study were to evaluate the association of serum pretreatment LMR, PLR, and RDW-SD with biopsy results in gray area patients and to develop an effective diagnostic nomogram for this population.

Inflammation is a key factor in the formation of a tumor microenvironment, and it also plays an important role in promoting tumor development^7^. Recently, it has been widely reported that a decreased pretreatment LMR is associated with unfavorable prognosis in patients with various malignancies^8–10^. However, few studies have reported the importance of LMR in differentiating malignancies from benign diseases. In our study, the median LMR value of the PB group was significantly lower than that of the NB group; this may have been caused by the increased count of CD14+ CD16+ monocytes, which have protumorigenic characteristics, in the PB group^11^. This finding corroborated that of a study performed by Volkan et al.^12^, in which gray area patients with PCa exhibited a significantly lower mean LMR value than gray area patients with benign prostatic hyperplasia (BPH). However, whether NLR can serve as an independent predictor of PB results in gray area patients remains controversial^12,13^. Huang et al.^14^ analyzed the data of 164 Chinese gray area patients and found that patients with NLR ≥ 2.44 exhibited a significantly higher PCa detection rate than those with NLR < 2.44 (36/77 vs 14/87; *P* < 0.001); they identified NLR as an independent predictor of transperineal biopsy results. However, using a larger Chinese sample, in the present study, we did not find an independent predictive value of NLR for PB results, even though it demonstrated significance in the univariate analysis. Additionally, although the mean PLR has been reported to be significantly higher in patients with PCa than in patients with BPH^15^, some Western studies have reached a consensus on the view that PLR cannot be considered as an independent predictor of PB results in gray area patients^12,16^. Our study is the first to analyze the data of Chinese gray area patients, identifying that pretreatment PLR has no independent predictive value for PB results.

RDW, a parameter reflecting the heterogeneity of erythrocyte volume, is reportedly helpful in discriminating malignant from benign biliary obstructions^17^ and in differentiating breast cancer from fibroadenomas^18^. Regarding prostate diseases, Albayrak et al.^19^ and Sun et al.^20^ reported that the mean RDW-coefficient of variation (CV) value exhibited by patients with PCa was significantly higher than that exhibited by healthy controls. Moreover, it was reported that RDW-CV exhibited by Chinese patients with PCa was significantly higher than that exhibited by patients with BPH and that high RDW-CV is an independent risk predictor of PCa^21^. Interestingly, a significant positive correlation was found between RDW-SD and RDW-CV in the current study, in which only patients with serum PSA levels between 4 and 10 ng/ml were included. However, high RDW-SD was found to be associated with low PCa detection rate, which is attributable to the following. First, BPH (accounting for a major proportion of the prostate diseases in the NB group in this study) was attributed largely to chronic inflammation^22^, and inflammatory cytokines can desensitize bone marrow erythroid progenitors to erythropoiesis, inhibiting red blood cell maturation and further resulting in the presence of newer and larger reticulocytes in the circulation^23,24^. Thus, the mean RDW-CV and RDW-SD values of patients with BPH are likely to be much higher than those of individuals without prostate (and hematological) diseases. Second, pretreatment RDW-CV value has an evidently positive correlation with serum PSA levels in patients with PCa^19,21^; therefore, the mean RDW-CV and RDW-SD values of gray area patients with PCa would, to a large extent, be lower than those of overall patients with PCa and may only be slighter higher than those of individuals without prostate (and hematological) diseases.

Among the 401 patients included in the study to construct the nomogram, 236 were from northwest China and 165 from other regions throughout China. Thus, our study cohort may be a good representation of the general population of Chinese gray area patients undergoing screening for biopsy. The present study is the first to evaluate the association of serum pretreatment LMR, PLR, and RDW-SD with biopsy results in Chinese gray area patients and to develop a nomogram to predict the individual risk of positive 13-core biopsy results exclusively in this group of patients. Furthermore, all the three variables contributing to our nomogram are inexpensive to attain in clinical practice, which guarantees the convenience of using this tool.

However, the present study does have several limitations that should be considered. First, prostate biopsies at our centers may be limited by the extent of sampling, and some patients with PCa may have been missed on initial biopsy, which may affect the accuracy of our conclusions. Second, magnetic resonance imaging findings of patients were not considered because the relevant information of many patients was unknown. Finally, patients in the validation cohort were from the same center as those in the training cohort, which may impair the universal applicability of our nomogram. Despite these limitations, our nomogram, established on existing conditions, performed well on both internal validation with bootstrapping and external validation using an independent cohort.

## Conclusions

To our knowledge, the present study is the first to report the importance of LMR and RDW-SD in screening for PCa among gray area patients who undergo 13-core biopsy and the first to construct a nomogram exclusively to predict the individual risk of PB results in this group. With its superior clinical utility, our nomogram is more accurate than PSAD, which was strongly recommended by the 2014 Chinese Urological Guidelines. Considering that PCa is a malignancy with several heterogeneities among patients of different races, validations using data of Western gray area patients are required to yield high-level evidence for the clinical application of our nomogram.

## Data Availability

The datasets are available from the corresponding author on reasonable request.
